# Prognostic role of the pre-treatment platelet-lymphocyte ratio in pancreatic cancer: a meta-analysis

**DOI:** 10.18632/oncotarget.20871

**Published:** 2017-09-14

**Authors:** Zheng-Shui Xu, Fa-Peng Zhang, Yin Zhang, Yong-Peng Ou-Yang, Xiao-Wen Yu, Wen-Long Wang, Wen-Ji Xu, Zhi-Qiang Luo

**Affiliations:** ^1^ Department of General Surgery, GuangRen Hospital of Xi’an Jiaotong University, 710000, Xi’an, Shaanxi, China; ^2^ Department of General Surgery, The Second Affiliated Hospital of Sun Yat-Sen University, 510120, Guangzhou, Guangdong, China; ^3^ Department of General Surgery, The Second Affiliated Hospital of Nanchang University, 330006, Nanchang, Jiangxi, China

**Keywords:** PLR, pancreatic cancer, overall survival, meta-analysis

## Abstract

**Background and Aims:**

Recently, the pre-treatment platelet-lymphocyte ratio (PLR), which is based on blood parameters, was accepted as a prognostic factor for patients with various cancers. Numerous studies have investigated the prognostic role of the PLR in pancreatic cancer; however, it remains unclear. Therefore, we conducted this meta-analysis to evaluate the relationship between the pre-treatment PLR and overall survival (OS) in pancreatic cancer.

**Materials and Methods:**

We performed a systematic literature search of the PubMed, Embase and Web of Science databases for relevant studies that explored the prognostic role of the pre-treatment PLR in pancreatic cancer. The hazard ratios (HRs) and 95% confidence intervals (CIs) related to OS were pooled using a random effects model.

**Results:**

Fourteen retrospective cohort studies involving 2,260 patients were included in this meta-analysis. Compared with low PLR, high PLR was a predictor of shorter OS (HR = 1.24, 95% CI: 1.10–1.39, I^2^ = 74%).

**Conclusions:**

In this meta-analysis, high pre-treatment PLR was a bio-predictor of short OS in patients with pancreatic cancer, suggesting that PLR could be used to predict prognosis of patients with pancreatic cancer before treatment. However, additional well-designed and large-scale studies are necessary.

## INTRODUCTION

Pancreatic cancer is the fifth most common cancer and ranks fourth in cancer-related mortality worldwide [1]. Although the mortality rate of pancreatic cancer is very high, histopathology and imaging remain the main methods used to evaluate prognosis in pancreatic cancer patients. Thus, progress in predicting prognoses remains unsatisfying, with no breakthroughs. Recently, many studies have described the role of the systemic inflammatory response in the development and progression of cancer [2–4]. Therefore, systemic inflammatory factors based on blood parameters, especially the neutrophil-lymphocyte ratio (NLR) and the platelet-lymphocyte ratio (PLR), are believed to be associated with the prognosis of patients with cancer. In fact, in the last year, many researchers have demonstrated the value of the PLR for predicting the prognosis of various cancers, such as lung cancer, esophageal cancer, gastric cancer and colorectal cancer [5–8]. However, could the PLR be applied to pancreatic cancer? Many studies have evaluated the relationship between high PLR and survival in pancreatic cancer. However the role of the PLR remains unclear. Therefore, we conducted this meta-analysis to evaluate the value of the pre-treatment PLR for predicting the prognosis of pancreatic cancer.

## MATERIALS AND METHODS

### Literature search

**Figure 1:**
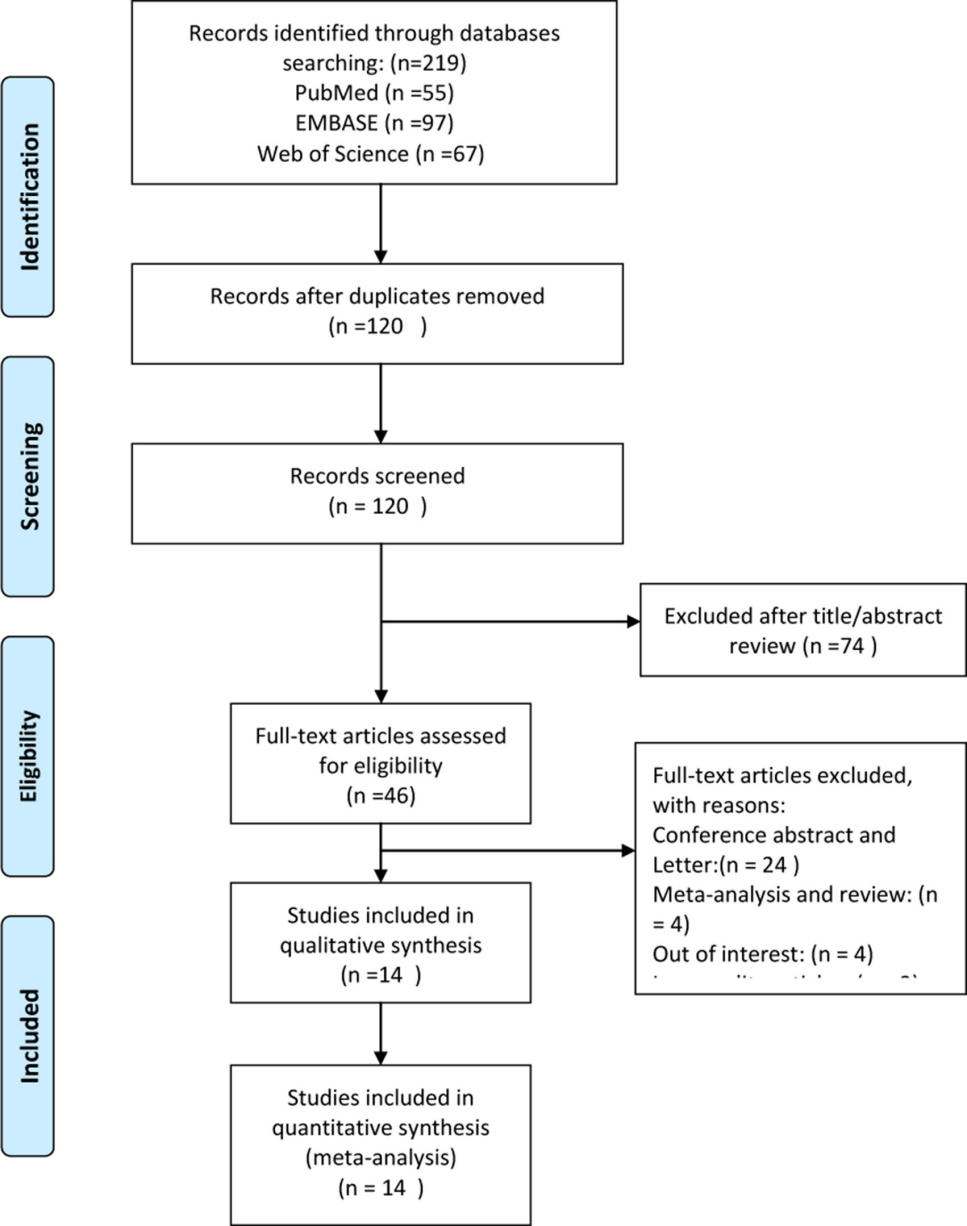
The flow chart of the study selection process

The present study was conducted according to the Preferred Reporting Items for Systematic Reviews and Meta-Analyses (PRISMA) statement [9]. A systematic literature search of the PubMed, Embase and Web of Science databases was performed for all studies published from inception to Nov. 23, 2016. To retrieve as many potential studies as possible, we performed an enlarged search strategy: (((“Pancreatic Neoplasms”[Mesh]) OR ((Pancrea*[Title/Abstract]) AND (((((((adenocarcinoma*[Title/Abstract]) OR tumour*[Title/Abstract]) OR tumor*[Title/Abstract]) OR neoplas*[Title/Abstract]) OR carcinoma*[Title/Abstract]) OR cancer*[Title/Abstract]) OR malignant[Title/Abstract])))) AND (((“platelet lymphocyte ratio”[Title/Abstract]) OR “platelet to lymphocyte ratio”[Title/Abstract]) OR PLR[Title/Abstract]). In addition, the references of relevant studies were carefully scanned to avoid missing any possible studies. All studies were independently categorized according to the pre-designed eligibility criteria. Any disagreements or questions were resolved by discussion or referral to a senior investigator. Figure 1 shows the flow chart of the study selection.

### Inclusion and exclusion criteria

We only included studies that described high pre-treatment PLR for pancreatic cancer compared with low PLR. The primary outcome was OS, and the included studies needed to report a hazard ratio (HR) and 95% confidence interval (CI) or include data that allowed those values to be calculated indirectly; otherwise, the study was excluded. The inclusion and exclusion criteria for this meta-analysis are presented using the Patients-Intervention-Control-Outcomes-Study designs (PICOS) form (Table 1).

**Table 1:** The detailed inclusion and exclusion criteria

	Patients	Intervention	Control	Outcomes	Study designs	Sampe size	Language	Follow-up
**Inclusion criteria**	Patients with pancreatic cancer	High PLR; The blood samples must be obtained before treatment	ow PLR; The blood samples must be obtained before treatment.	OS with the HR and 95%CI	Control studies or randomized controlled trials	Any	Any	Any
**Exclusion criteria**	Patients with pancreatic cancer	High PLR; The blood samples did not be obtained before treatment.	Low PLR; The blood samples could not be obtained before treatment.	Without OS or its value of HR and 95% CI could not be collected by the original article	letters, conference abstracts, review articles, descriptive studies	No	No	No

PLR = platelet lymphocyte ratio; OS = overall survival; HR = hazard ratio; CI = confidence intervals.

### Data collection and assessment of methodological quality

All the relevant information was collected in our pre-designed table:

Patients (P): country, age, sample size, histology and stage of pancreatic cancer, and type of treatment.

Intervention (I): the group with high PLR.

Control (C): the group with low PLR.

Outcomes (O): the definition of OS and the data for HR and 95% CI.

Study designs (S): the type of study design, the details used for patient selection, the comparability of the study groups and the assessment of outcome.

The Newcastle–Ottawa Scale (NOS) was used to assess the quality of each study [10]. Studies with an NOS score ≥ 6 were considered high quality; all others were considered low quality and were not included.

### Statistical analysis

The pooled outcome was evaluated using the HR and 95% CI values. The HR represents the hazard of OS in the high PLR group compared with that in the low PLR group. HR values greater than 1 implied poor OS for the high PLR group, and the OSs of the high PLR group and the low PLR group showed statistically significant differences when the pooled HR and the 95% CI did not include the value 1. Because all the included studies were retrospective studies and potential differences between them should be taken into account, the inverse variance method was used to pool the HR for OS using a conservative random effects model [11]. In addition, the I^2^ statistic was applied to evaluate the heterogeneity of the included studies. I^2^ < 50% suggested that there was no significant heterogeneity across the included studies and was deemed acceptable [12]; otherwise, we would have performed post hoc subgroup analysis to investigate the potential heterogeneity across the included studies according to sample size (<200 versus >200), cut-off values (<200 versus >200), different therapeutic modalities (operation VS no-operation) and stage (I/II versus III/IV). To validate the credibility of the pooled outcome, we conducted an influence analysis using the “metainf” STATA command; this process omitted one study each time. Publication bias was evaluated using visual inspection of funnel plots and Egger [13] and Begg's [14] tests. All statistical tests included a bilateral *P* value, and *P* values < 0.05 were considered statistically significant. RevMan 5.3 (the Nordic Cochrane Centre, the Cochrane Collaboration) and Stata 12.0 (StatCorp, College Station, TX, USA) were used to perform all statistical analyses.

## RESULTS

A total of 219 records were acquired from the three databases (PubMed, Embase and Web of Science) through our expanded search strategy. After duplicate and irrelevant records were removed, 46 potentially eligible studies remained. The full texts of the remaining studies were checked for other possible studies. Finally, 14 retrospective cohort studies involving 2,260 patients were included in this meta-analysis [15–28].

### Characteristics of the included studies

We included 14 retrospective cohort studies in this meta-analysis (Table 2). Sample sizes ranged from 37 to 386, and the cut-off values used in the studies ranged from 126 to 300. HRs with corresponding 95% CIs were directly reported in all included studies, 8 of which calculated HRs using univariable analysis [16, 17, 19, 20, 23, 25–27] and 6 using multivariate analysis [15, 18, 21, 22, 24, 28].

**Table 2:** Characteristics of all the studies included in the meta-analysis

Reference, year	Country	Ethnicity	Age^*^	No.Sample	Design	Stage	Histology	Treatment	Cut-off	NOS
Alagappan M 2016	Asari S 2016	Bhatti I 2010	Inoue D 2015	Kishi T 2015	Lee J M 2016	Liu Z 2016	Martin H L 2014	Qi Q 2015	Shirai Y 2015	Smith R A 2009	Tao L 2016	Wang D S 2012	Watanabe J 2016
USA	Japan	UK	Japan	Japan	Korea	China	Australia	China	Japan	UK	China	China	Japan
Caucasian	Asian	Caucasian	Asian	Asian	Asian	Asian	Australoid	Asian	Asian	Caucasian	Asian	Asian	Asian
75.2(65.9–86.1)	68(60.3–73)	65(51–79)	67(32–88)	65 (35–85)	63.5 ± 10.7	61(34–83)	68.5 (35–90)	61.2 ± 10.7	66.5 ± 10.2	67 (61–73)	63.4(23–86)	NR	67(32–88)
208	37	84	440	65	82	386	124	211	131	110	159	177	46
Retrospective	Retrospective	Retrospective	Retrospective	Retrospective	Retrospective	Retrospective	Retrospective	Retrospective	Retrospective	Retrospective	Retrospective	Retrospective	Retrospective
III/IV	I-II	NR	I-IV	III/IV	III/IV	I-IV	III/IV	III/IV	I-II	I-II	NR	Ib-IV	Ia-IIb
PDAC	PDAC	PDAC	PDAC	PC	PDAC	PDAC	PC	PDAC	PDAC	PDAC	PDAC	PDAC	PDAC
R/C	O/C	O/C	O/C/P	CR	C	NR	R /C/P	C	O	O/C	O	O/C/R	O/C O/C
200	225	200	150	150	150	165.5	200	126	150	300	130.96	300	200
6	8	9	7	8	7	7	7	6	7	8	7	8	7

^*^values are mean(s.d.) or mean range or median (range) or median(interquartile range) values without s.d. or range are means.

C = chemotherapy, O = operation, R = radiotherapy, P = palliative care, HR = hazard ratio, PLR = platelet-to-lymphocyte ratio, PDAC = pancreatic ductal adenocarcinoma, PC= pancreatic cancer, OS = overall survival, NOS = Newcastle-Ottawa Scale, NR = not reported.

### Outcome

**Figure 2:**
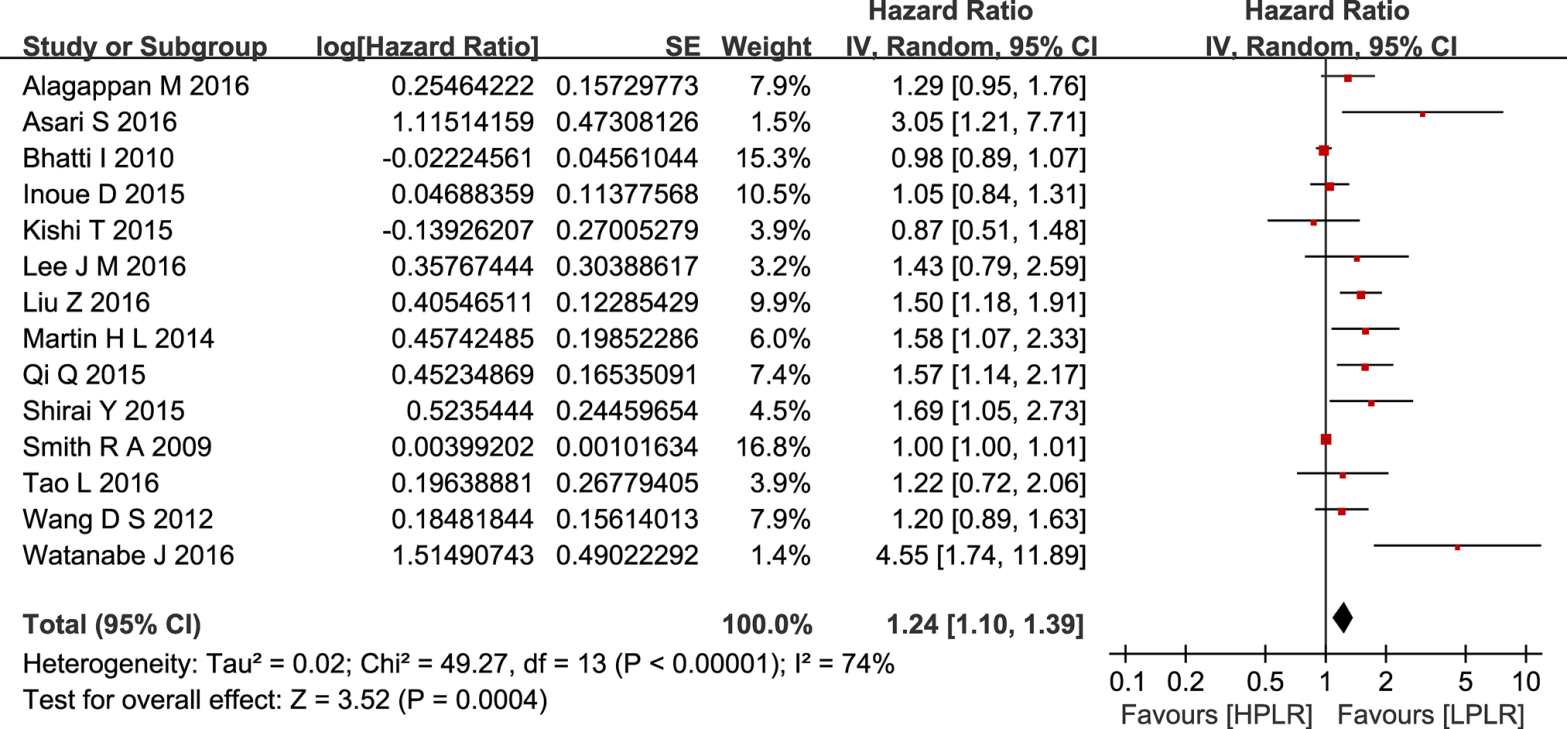
Forest plots of included studies evaluating the hazard ratio of overall survival SE = standard error, CI = confidence interval, IV = inverse variance.

**Figure 3:**
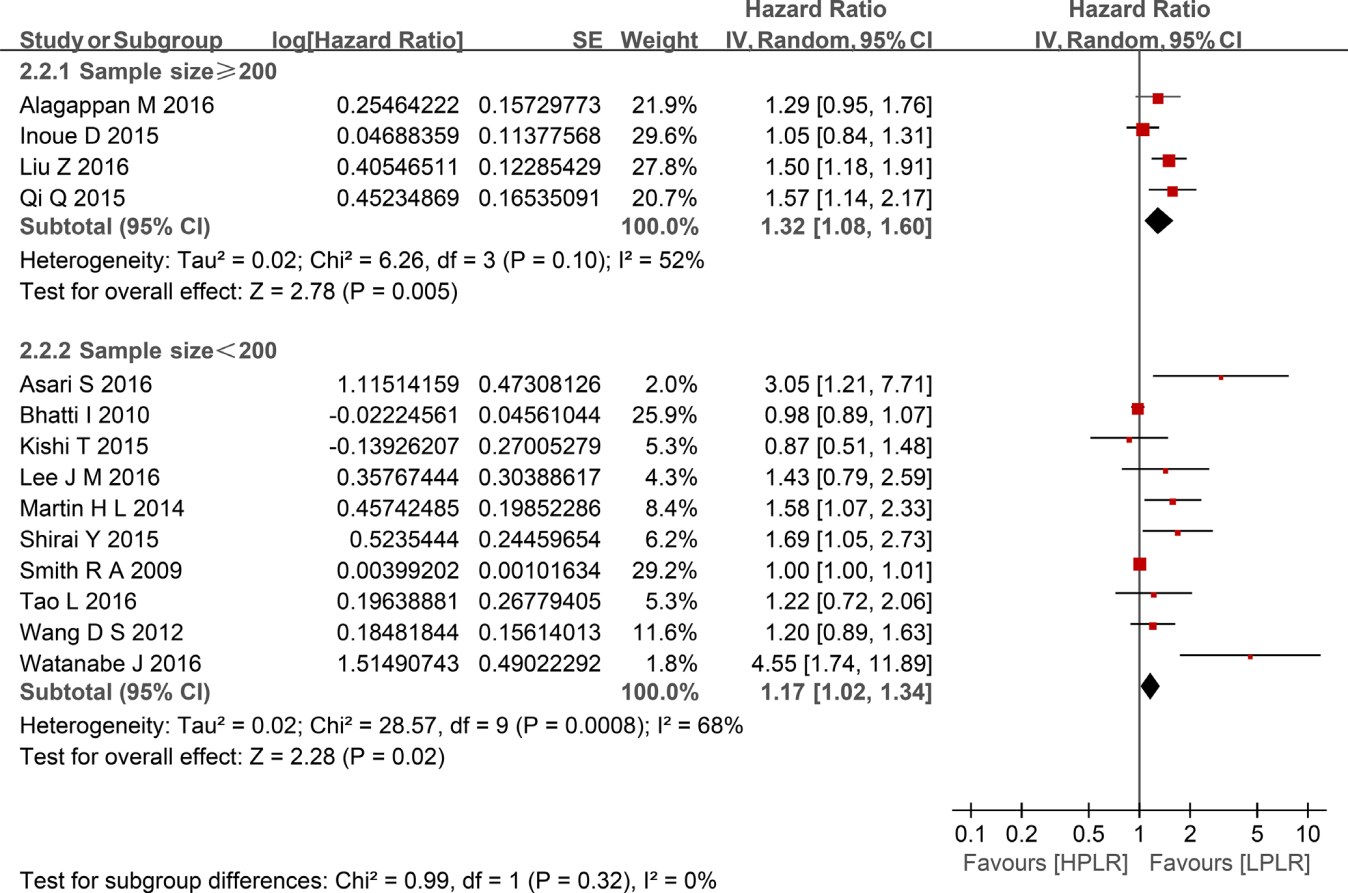
Forest plots of HPLR versus LPLR with OS in subgroups of sample size SE = standard error, CI = confidence interval, IV = inverse variance.

**Figure 4:**
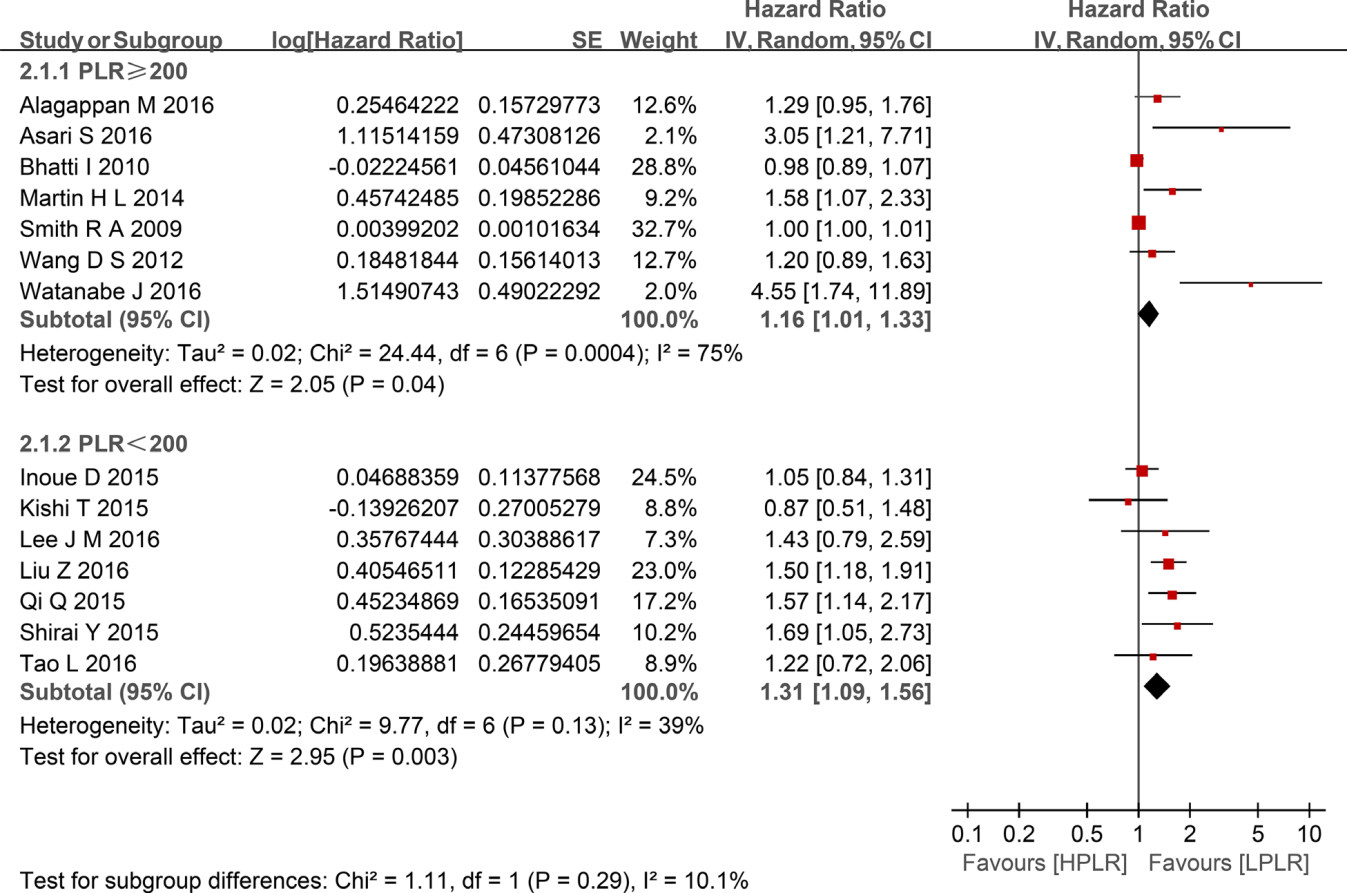
Forest plots of HPLR versus LPLR with OS in subgroups of cut-off for PLR SE = standard error, CI = confidence interval, IV = inverse variance.

**Figure 5:**
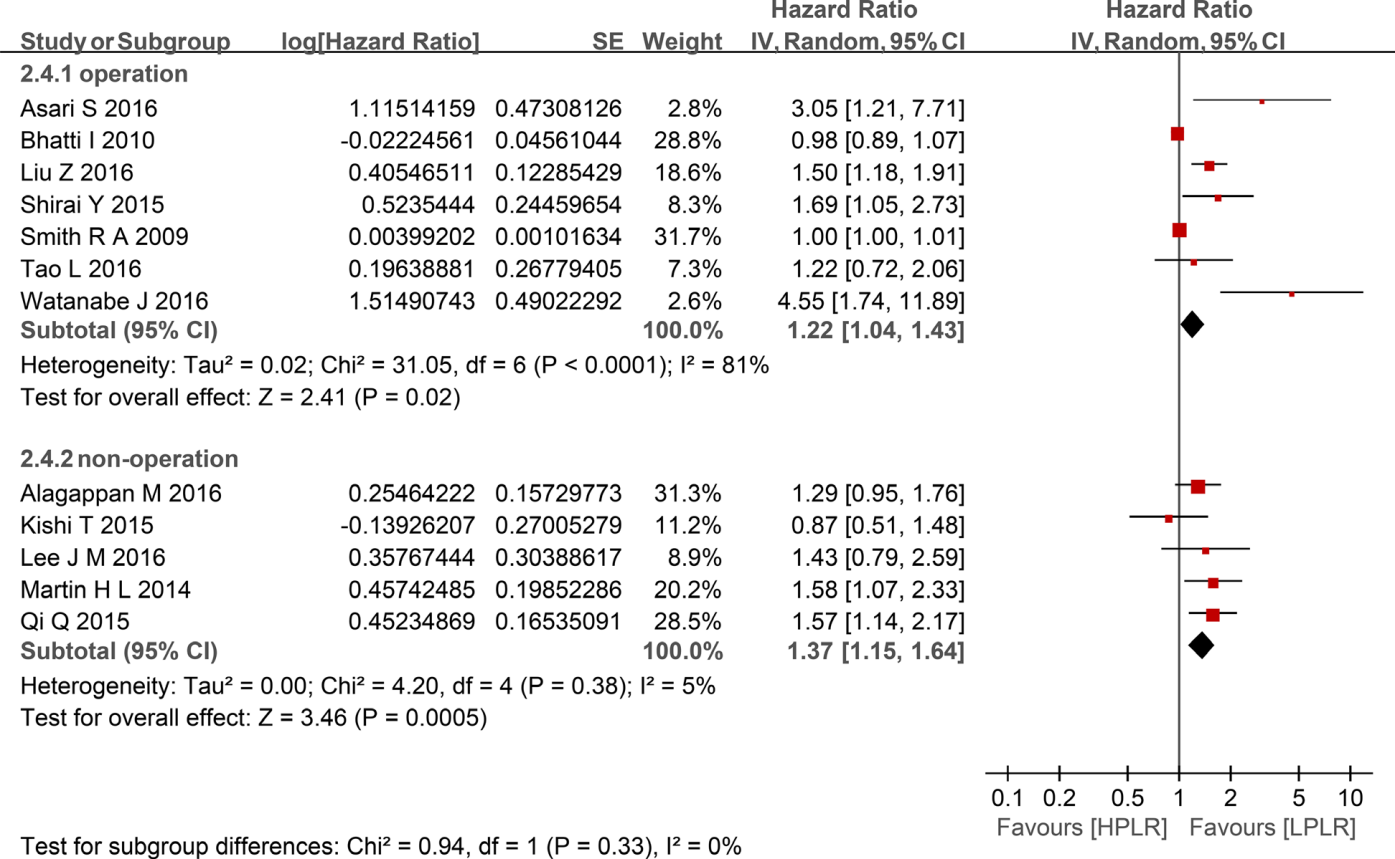
Forest plots of HPLR versus LPLR with OS in subgroups of treatment SE = standard error, CI = confidence interval, IV = inverse variance.

**Figure 6:**
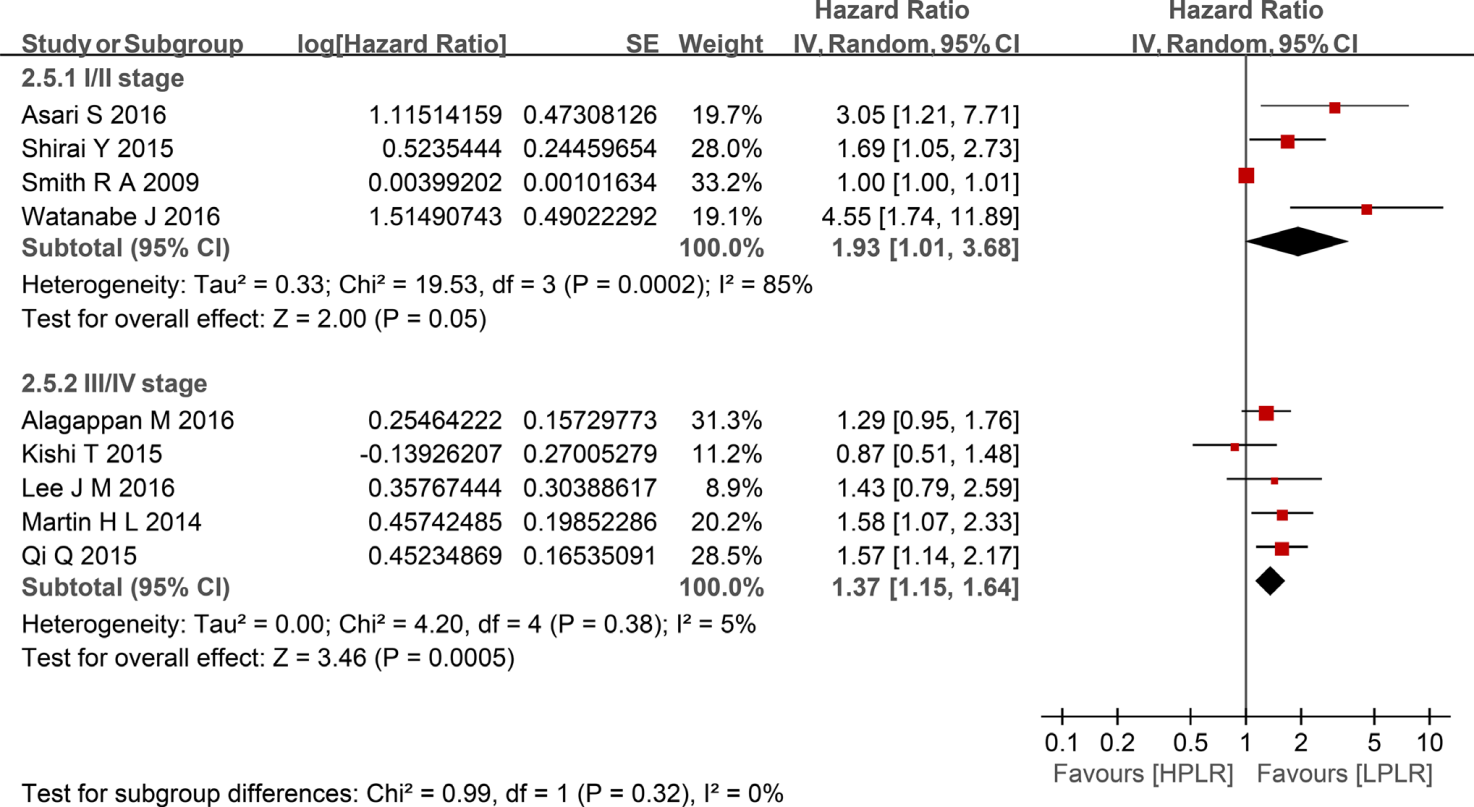
Forest plots of HPLR versus LPLR with OS in subgroups of stage SE = standard error, CI = confidence interval, IV = inverse variance.

**Figure 7:**
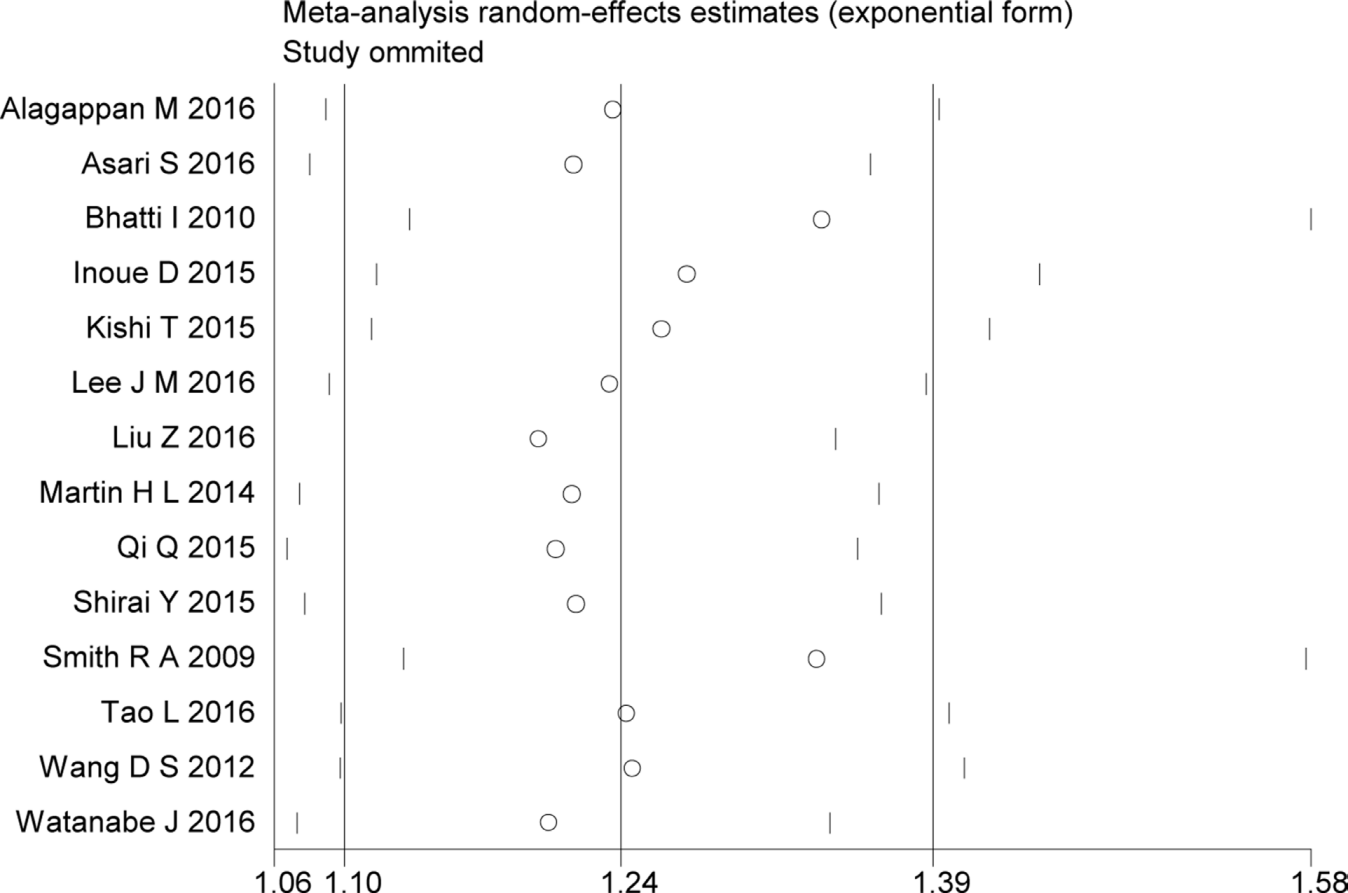
Sensitivity analyses of the included studies evaluating the hazard ratio of overall survival SE = standard error, CI = confidence interval, IV = inverse variance.

Compared with low PLR, elevated PLR was a predictor of shorter OS (HR = 1.24, 95% CI: 1.10–1.39, I^2^ = 74%; Figure 2). The subgroup analyses demonstrated no potential heterogeneity because of sample size (Figure 3), cut-off value (Figure 4), different therapeutic modalities (Figure 5) or stage (Figure 6). We also conducted a sensitivity analysis to validate the credibility of the pooled outcomes. When we removed any study one at a time, the pooled outcome was not markedly impacted (Figure 7).

### Publication bias

**Figure 8:**
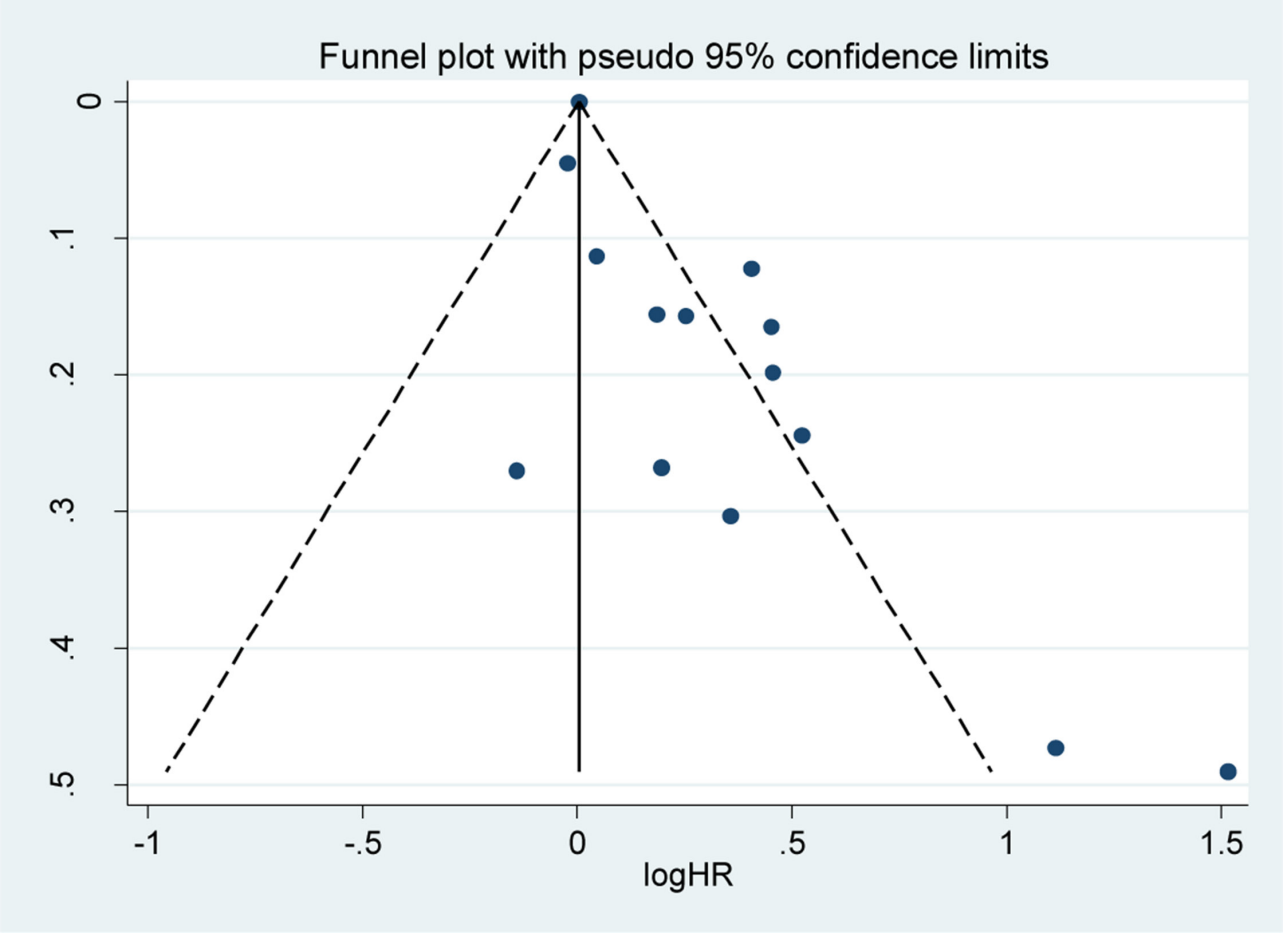
Funnel plot for the assessment of potential publication bias SE = standard error, HR = hazard ratio. SE = standard error, CI = confidence interval, IV = inverse variance.

The funnel plot seemed to be asymmetrical upon visual inspection, but publication bias was not detected using the statistical tests of Egger (*P* = 0.10) and Begg (*P* = 0.10; Figure 8).

## DISCUSSION

Recently, some researchers have suggested that the interaction between platelets and cancer is reciprocal; in other words, tumors can stimulate platelet activity and production, while platelets can promote tumor growth, invasion and metastasis [29, 30]. Although “it is difficult to distinguish between a mere correlation with platelets and cancer and an actual causality” [29], it is accepted that high platelet counts are a predictor of poor prognosis in cancer [29, 31–35]. In addition, lymphocytes play a crucial role in lymphocyte-mediated anti-tumor activity by “inducing cell apoptosis and inhibiting cancer cell proliferation and migration;” thus, lymphocytopenia would weaken this role without question [4, 36]. A high PLR accompanies either thrombocytosis or relative lymphocytopenia, both of which seem to be harmful to patients with cancer. Many researchers have demonstrated that a high PLR is a negative predictor of prognosis in various cancers, such as lung cancer, esophageal cancer, gastric cancer and colorectal cancer [5–8]. Many studies have also been performed to evaluate the relationship between PLR and survival in pancreatic cancer, but the results have been inconsistent. Among these studies, two meta-analyses showed that a high PLR was associated with poor OS in various cancers, although in the subgroup of pancreatic cancer patients, PLR showed no association with OS in these meta-analyses, which both only included 3 studies involving several hundred patients [37, 38]. Thus, the role of the PLR in pancreatic cancer remains uncertain, and we conducted the current meta-analysis including 14 studies and 2,260 patients to address these previous inconsistencies. Besides that, we made more rigorous inclusion and exclusion criteria, for example we only included patients before any anti-cancer treatment which can influence the blood parameters. And we perform subgroup analysis and influence analysis to validate the credibility of the pooled outcome in this meta-analysis. So we made a more scientific conclusion.

This meta-analysis included 14 retrospective cohort studies involving 2,260 patients and demonstrated that a high PLR was a better predictor of shorter OS than a low PLR, with an HR of 1.24 (95% CI: 1.10–1.39, I^2^ = 74%). Additionally, subgroup analysis did not indicate a significant difference between studies with sample sizes < 200 and those ≥ 200. Given the various cut-off values of the PLR in the included studies, a subgroup analysis based on cut-off values (< 200 versus ≥ 200) was also performed, and we found that the high PLR group had a shorter OS than the low PLR group, regardless of cut-off value used. So did the subgroup analysis of different therapeutic modalities (operation VS no-operation) and stage (I/II versus III/IV). To validate the credibility of the pooled outcome, we performed an influence analysis using the “metainf” STATA command; it proved that no one study obviously impacted the pooled outcome of interest. Although the heterogeneity could not be explained, these results strengthen the possibility that a high PLR is associated with a short OS in patients with pancreatic cancer. However, it is possible that the included studies that did not have robust control for confounders actually diluted the value of the PLR for the prognosis of patients with pancreatic cancer. We hypothesized that the potential heterogeneity may have been derived from clinical factors, such as mixed treatment, the stratification of different stages of pancreatic cancer, and the inadequacy of follow-up, although these factors could not be analyzed in the present study.

Several suggestions can be made regarding the further development of the PLR as a bio-predictor. First, we should control for the influence of several factors that may influence platelet counts, such as the patient's basic state, the presence of infection or diseases and drug treatment, to draw more rigorous scientific conclusions. Second, future original studies should compare more outcomes, such as tumor diameter, lymph node metastasis, stage, distant metastasis, local recurrence, and disease-free survival, between high and low PLR groups. These comparisons may indirectly demonstrate the relationship between PLR and pancreatic cancer. Third, adequate follow-up is necessary. Fourth, we should pay more attention to the change in PLR between pre-treatment and post-treatment protocols, which may provide another way to assess the therapeutic efficacy and the patients’ prognosis. With such developments, the PLR may represent an inexpensive and simple bio-predictor for future use.

### Limitations

First, multiple PLR cut-off values were applied in the studies included in this meta-analysis. Although the subgroup analysis did not indicate that there were significant differences between cut-off values of < 200 and > 200, it is unclear which PLR cut-off value should be applied clinically. Second, PLR measurements based on blood parameters can be influenced by the patient's basic state, infection or disease and drug treatment. Third, although no publication bias was detected, the potential for it cannot be excluded. Finally, the obvious heterogeneity of the studies cannot be ignored. The potential heterogeneity that may derive from uncontrolled or unmeasured risk factors, such as mixed treatment, the stratification of different stages of pancreatic cancer and inadequate follow-up, need to be further evaluated in the future. Furthermore, additional well-designed and large-scale studies are necessary to demonstrate the value of PLR in pancreatic cancer and establish a more precise cut-off value for clinical applications. Thus, the conclusions of this study should be interpreted with caution.

## CONCLUSIONS

High pre-treatment PLR is a bio-predictor of short OS in patients with pancreatic cancer. Given these findings, the PLR might be applicable for predicting the prognosis of patients with pancreatic cancer before treatment. However, additional well-designed and large-scale studies are necessary.
